# Safe Babies, Safe Moms: A Multifaceted, Trauma Informed Care Initiative

**DOI:** 10.1007/s10995-023-03840-z

**Published:** 2023-11-20

**Authors:** Loral Patchen, Asli McCullers, Charmain Beach, Melanie Browning, Shy Porter, Aimee Danielson, Evelyn Asegieme, S. Roxana Richardson, Ali Jost, Caitlin Schille Jensen, Naheed Ahmed

**Affiliations:** 1 Women and Infant Services, MedStar Washington Hospital Center, 110 Irving St NW, Washington, D.C., DC 20010 USA; 2 MedStar Center for Health Equity Research, MedStar Health Research Institute, 6525 Belcrest Rd #700c, Hyattsville, MD 20782 USA; 3 Department of Psychiatry, MedStar Georgetown University Hospital, 2115 Wisconsin Ave NW, 2nd Floor, Washington, DC, 20007 USA; 4 Georgetown University Health Justice Alliance Perinatal Legal Assistance and Well-being Project, 600 New Jersey Avenue NW, Washington, DC, 20001 USA; 5 LLC, Washington, D.C., USA; 6 NYU Grossman School of Medicine, 180 Madison Avenue, New York, NY 10016 USA

**Keywords:** Health Disparities, Perinatal Mental Health, Medical-legal Partnership, Trauma Informed Patient care Model

## Abstract

**Purpose:**

This report describes a multifaceted, trauma-informed initiative developed to address racial/ethnic maternal and infant health inequities in Washington, D.C.

**Description:**

Structural racism and systemic oppression of marginalized communities have played a critical role in maternal and infant health inequities in the United States. Black birthing individuals are exponentially more likely to experience adverse birth outcomes, including preterm birth, low birth weight and maternal mortality. In response to these statistics, the Safe Babies Safe Moms (SBSM) initiative was developed to support patients of marginalized identities and improve health outcomes. SBSM Women’s and Infants’ Services Specialty Care (WIS-SC) is one component of this initiative focused on perinatal services.

**Assessment:**

SBSM WIS-SC includes trauma-informed clinical services, nurse navigation, lactation, diabetes and nutrition education, social work services, medical-legal services, and behavioral health support. Services are delivered by a multidisciplinary team trained on the following domains: (1) building connection within diverse care teams; (2) recognizing systemic barriers to trauma-informed approaches; (3) learning the brain science of implicit bias, trauma, and resilience; (4) Integrating self-care practices; and (5) acknowledging progress. Since the inception of the program, SBSM WIS-SC has served over 1500 patients.

**Conclusion:**

The SBSM WIS-SC intervention reflects a patient-centered approach to care, offering the multidisciplinary services required for perinatal patients with complex medical, psychosocial, and legal needs. Trauma informed training and team building is foundational to successful service delivery to address these multifaceted health needs of historically marginalized perinatal populations nationwide.

## Significance

*What is Already Known on this Subject?*: The consequences of structural racism have a profound impact on infant and maternal health outcomes among racial and ethnic minorities. Trauma informed, patient centered care models are integral to dismantling such disparities within healthcare systems.

*What does this Study adds?*: This from-the-field article provides an in-depth glance at the workings of Safe Babies Safe Moms Women’s and Infants’ Services Specialty Care (SBSM WIS-SC), a Washington D.C. based initiative designed to serve as a comprehensive and multifaceted intervention to identify, support, and connect perinatal patients of marginalized identities to trauma-informed resources.

## Introduction

There is an urgent need to address the complex role that structural racism plays in adverse maternal and child outcomes among historically marginalized racial/ethnic women. Structural racism, defined as “forms of racism that are pervasively and deeply embedded in and throughout systems, laws, written or unwritten policies, entrenched practices, and established beliefs and attitudes that produce, condone, and perpetuate widespread unfair treatment of people of color” has led to deleterious health outcomes among marginalized racial/ethnic populations (Braveman et al., 2022; Gillespie-Bell, 2021). This oppression has led to patients of marginalized racial identities not only facing limited access to critical healthcare resources but also poorer quality of healthcare services (Gillespie-Bell, 2021; Fernandez et al., 2021; Crear-Perry et al., 2002; Leonard et al., 2019). Maternal and child health outcomes, which are sensitive to stress and reflect the impact of structural racism, are worse among minority racial groups, in particular Black birthing individuals, in relation to preterm birth, low birth weight, and maternal mortality rates. Black birthing individuals are 2–6 times more likely to die during childbirth in comparison to other racial/ethnic groups (Flanders-Stephans, 2000; Sutton et al., 2020). These alarming statistics underscore the urgency of designing and implementing comprehensive healthcare services to address these significant disparities in maternal and child health.

## Description

Safe Babies Safe Moms (SBSM) is a comprehensive and multifaceted initiative within a reginal health system designed to address poor maternal and child health outcomes in Washington, DC (Medstar Health, 2023). SBSM Women’s and Infants’ Services Specialty Care (WIS-SC) is a component of this initiative focused on the perinatal period and maternal outcomes. SBSM WIS-SC includes clinical services, nurse navigation, social work services, diabetes and nutrition education, lactation, medical-legal services, and behavioral health support. Service delivery is informed by a universal comprehensive assessment tool that includes validated questions and tools to identify support needed across multiple domains to better understand the needs and priorities of patients. These domains include social services intervention, health education, health harming legal needs, clinical care coordination, and behavioral health. Validated instruments included in the assessment are the Edinburgh Postnatal Depression Screen (Cox et al., 1987) and adapted from the first ten questions of the Centers for Medicare & Medicaid Services Accountable Health Communities’ Health-Related Social Needs Screening Tool (AHC-HRSN, 2017). The assessment is conducted during triage for office appointments at three points on the care continuum: the initial obstetric appointment; the third trimester between 24 and 28 weeks; and at the 6-week postpartum visit. A nurse navigator completes the initial and postpartum assessment with the patient, and either a medical assistant or nurse completes it during the third trimester.

The majority of people served by the SBSM initiative (84.2%) are Black or African American making them vulnerable to healthcare racism; and most (56.3%) live in Washington, D.C. wards 5, 7, or 8, which are underserved and under-resourced areas. A higher-than-average proportion of pregnant people who have public insurance (94.5% compared to 51.0% nationally) are served by the initiative, indicating a high number of patients who are of lower socioeconomic status. 15.2% of SBSM WIS-SC patients had a history of preterm delivery, compared to the national average of 10% of babies being born prematurely. See Table 1 for more descriptive information.

**Table 1:** Table one provides descriptive information collected at the first prenatal appointment for people served in this office

		Frequency	Valid %
Age	Teens (< 20)	56	9.1%
	Adults (20–34)	463	75.0%
	Advanced maternal age (> 34)	98	15.9%
Race	African American	521	84.2%
	Non-African American	85	13.7%
		13	2.1%
Primary language	English	318	92.4%
	Non-English	24	7.0%
	Unknown	2	0.6%
Employment/in school	Yes	154	46.7%
	No	110	34.1%
	No due to COVID-19	15	4.6%
	Unknown	44	13.6%
Marital status	Married	39	11.3%
	Unmarried	301	87.0%
	Unknown	6	1.7%
Insurance type	Public	583	94.5%
	Non-public	34	5.5%
Zip code	Wards 5, 7, or 8	351	56.3%
	Not wards 5, 7, or 8	272	43.7%
Received food insecurity support	Yes	86	32.8%
	No	176	67.2%
History of prior preterm birth	Yes	92	15.2%
	No	511	84.6%
	N/A or unknown	1	0.2%
History of low birth weight infant	Yes	57	9.4%
	No	546	90.4%
	N/A or unknown	1	0.2%
Gestational age at intake	Before 28 weeks	528	85.3%
	At or after 28 weeks	91	14.7%
One or More of: chronic hypertension (HT), gestational HT, preeclampsia, eclampsia, HELLP, or superimposed preeclampsia	Yes	142	23.5%
	No	463	76.5%
One or More of: diabetes mellitus, prediabetes, or gestational diabetes	Yes	67	11.1%
	No	536	88.9%

SBSM WIS-SC leadership recognized that offering services and intervention would only be effective if the patient’s experience in the care environment were also improved. To achieve the goal of transforming care delivery, leadership organized group team meetings to build a trauma-informed workforce. A trauma-informed approach is an organizational structure and treatment framework that involves understanding, recognizing, and responding to the effects of all types of traumas by embedding six principles -- safety, trustworthiness and transparency, peer support and mutual self-help, collaboration and mutuality, empowerment, voice and choice, and cultural issues -- into practice and services (SAMHSA, 2014). Organizations that successfully integrate trauma-informed practices are better able to sustain the workforce, foster resilience among patients and staff, and support the inevitable challenges transformation goals present to large healthcare systems. Given that SBSM WIS-SC with its innovative collaborative care goals, launched early into the global COVID-19 pandemic and a national reckoning with racial injustice, leadership sought to build shared language on trauma, its impact on workers and patients, and best practices to foster individual and team resilience. This trauma-informed lens dovetails with goals to address healthcare disparities, recognize the day-to-day impact of implicit bias on workers and patients, and facilitate a shift from the traditional deficit-based lens of “what is wrong with you” to a more grounded approach of “what happened to you.“

Training was considered foundational to achieving program goals as the SBSM WIS-SC team felt *how* services were delivered was as important as *which* services were delivered. Team development on trauma-informed practices included:

- Building connection: Taking space to help the care team recognize and value the complex roles played by each of its diverse team members.
- Recognizing systemic barriers to trauma-informed approaches: Learning to identify system challenges to building health equity and exploring ways to address barriers;
- Learning the brain science of implicit bias, trauma, and resilience: Recognizing how workers and patients are triggered by our day-to-day experiences delivering health care and adopting protective factors and practices to resist the frequency of implicit bias and facilitate regulation and collaboration.
- Integrating self-care practices: Recognize the need to take care of ourselves and to socialize outside of direct clinic work so that we can connect and take care of each other.
- Acknowledging progress: Using evaluation data to assess the compassion resilience of the SBSM WIS-SC workforce and taking time to recognize wins around change goals.

## SBSM WIS-SC Supports

The SBSM WIS-SC assessment facilitates conversations about patient priorities, goals, and care plans. Supports are aligned with the patient’s preference and needs. Not all interventions are needed or desired, and engagement with specific services is responsive to patient direction and evolves across the perinatal continuum. Patients received services based on reccomendations from the care team, follow-up from assessment, and patient request. Table 2 summarizes SBSM WIS-SC services.

**Table 2:** Summary of services offered through WIS-SC safe babies safe moms

Program	Program Description
Nurse Navigation Activities and Services	•Administer and review comprehensive assessments of social determinants of health 3 times during the perinatal period, providing appropriate linkages to care for identified needs such as food insecurity, baby supplies, WIC, etc.
	•Review clinical care plan with patient and provides education on self-management.
	•Resolve barriers to care, such as obtaining pre-authorizations for medications and arranging transportation.
	•Partner with healthcare provider to coordinate care needs and adherence to management plan.
	•Conduct health education groups, including childbirth, lactation, and newborn care.
Diabetic Management and Nutrition Education	•Offer general nutrition during pregnancy class monthly for all patients after their initial prenatal appointment.
	•Meet with patients referred by providers for consultation on guidelines for optimal gestational weight gain and remaining on target as well as special nutritional support services, such as management of Pica.
	•Provide specific diet education based on dietary recall and patient preferences.
	•Review blood sugar log and monitors for both diet and insulin modifications to optimize glucose management.
	•Conduct follow-up after delivery to provide education and coordination for follow-up with endocrinology and other services as needed.
P-Law Activities and Services	•Assess incoming patient referrals and determine appropriate action.
	•Conduct legal intakes with patients referred by WIS.
	•Provide direct legal assistance (including advice, brief service, and extended representation) to patients/clients referred by WIS.
	•Facilitate referrals for patients to other legal and social services providers when unable to take a patient’s case.
	•Collaborate with healthcare partners to advance client representation.
	•Conduct trainings for WIS healthcare providers, including on the MLP model, types of cases to refer, and areas of law impacting patients.
	•Conduct know-your-rights trainings for WIS patients and WIS providers/partners.
Social Work Services	•Respond to elevated depression and anxiety scores, supporting inpatient care as appropriate.
	•Conduct psycho-social assessments to determine alignment with therapeutic and psychiatry services.
	•Facilitate groups to prevent onset of mood disorder during the perinatal period using evidenced based intervention.
	•Facilitate cognitive behavioral group for people with postpartum onset of depression.
	•Collaborate with healthcare partners to advance mental health.
	•Facilitate knowledge of and access to social service interventions and community resources.
	•Provide brief, solution-oriented individual therapy (generally not to exceed 3 sessions).
Behavioral Health Services	•Offer initial assessment with family therapist after referral from social work team to determine therapeutic goals and if aligned with 8 session therapy.
	•Provide individual therapy as determined from initial assessment.
	•Provide co-located psychiatry services focused on medication management.

### Nurse Navigation, Lactation Support, and Childbirth Education

The nurse navigation team is responsible for the coordination of patient care across the continuum under the auspices of a provider’s prescribed plan of care. They also provide complex obstetric patients with specialized education, patient advocacy, follow-up support, and coordination of care throughout the duration of their pregnancy and postpartum period. Nurse navigation in the SBSM WIS-SC program is organized around clinical bundles, or medical conditions, associated with poor maternal and infant outcomes, such as hypertension and preterm birth. In addition, they conduct ongoing assessments and re-revaluation of social drivers of health to ensure needs are addressed. As part of the SBSM WIS-SC initiative, nurse navigators facilitate group education in lactation, newborn care, and childbirth education. Nurse navigators also play a vital role in promoting shared decision making within the care team, centering patients’ knowledge, preferences, and decisions in the clinical management plan.

### Diabetes Management and Nutrition Education

The perinatal diabetes and nutrition care team assists patients with diabetes in overcoming social, emotional, behavioral, and financial barriers to optimal self-management. At the first prenatal appointment, patients are encouraged to attend the nutrition during pregnancy class to reduce the occurrence of inadequate or excessive gestational weight gain, and reduce the risk of acquiring gestational diabetes and preeclampsia. The team applies evidence-based practice to assess and address several self-care behaviors, including healthy eating, physical activity, monitoring blood glucose, taking medication, problem solving barriers to self-care, and healthy coping. Barriers to self-care are identified at both the initial and all follow up appointments, and referrals are generated to the appropriate SBSM WIS-SC teams as desired by the patient (i.e., social work, behavioral health, nurse navigation, or P-LAW attorney). Services continue during the postpartum period to address concerns with blood glucose, blood pressure control, and weight management. Patients also are connected to endocrinology or primary care after delivery.

### Medical Legal Partnership

The Perinatal Legal Assistance & Well-being (P-LAW) project provides no-cost legal services to pregnant and postpartum patients grappling with unmet civil legal needs that interfere with their efforts to achieve optimal health and well-being for themselves and their infants. Most legal services focus on employment, housing conditions, and benefits. This medical-legal partnership (MLP) provides direct legal services to pregnant patients and newborns, education and training to students and providers, engages in community outreach and education, and contributes to the data collection and research to evaluate outcomes.

### Perinatal Social Work

Perinatal social workers (SW) assist patients in their effort to become successful parents and individuals by promoting psychosocial well-being and facilitating access to resources through social service interventions and therapeutic supports. The team conducts follow-up assessments to patients with identified needs either by self-referral or areas identified during SBSM WIS-SC assessment. Social workers facilitate a psychoeducational group to prevent perinatal mood and anxiety disorders for patients experiencing distress during the prenatal period that does not rise to the level of depression or anxiety. They also facilitate a cognitive behavioral postpartum group for mothers with postpartum onset of depressive symptoms. In addition, they offer brief, solution-oriented interventions to patients focused on an immediate need when identified in the clinical setting.

### Behavioral Health

The integrated behavioral health clinicians are responsible for providing mental health care via time-limited psychotherapy and psychiatric medication management to patients who screen positive for behavioral health needs. The SBSM WIS-SC integrated behavioral health team consists of social workers with specialized clinical and research training in perinatal mental health and referral to a psychiatrist with specialized training in reproductive psychiatry. It is not uncommon for the patients who receive medication management under the care of the psychiatrist to also be referred to time-limited psychotherapy and vice versa; thus, many patients are engaged in combined treatment with psychotherapy and psychiatric medication which is consistent with best practice and evidence-based care. The two clinicians work closely in collaboration with one another, as well as with the midwife/physician and other SBSM WIS-SC team members to consult on cases and ensure patients’ mental health needs are appropriately met.

## Conclusion

The SBSM WIS-SC intervention reflects a patient-centered approach to care, which integrates services required for the perinatal care of patients with complex medical, psychosocial, social, environmental, and legal needs. Comprehensive and iterative team training in trauma-informed practice established a culture that elevated the experience of care as paramout, underscoring that how people received care in SBSM WIS-SC was as important as the service delivered. This trauma-informed care model shifts the onus from patients and providers to collaborative teams to identify and address barriers to care among marginalized patient populations. Recognizing the varied factors that affect health, SBSM WIS-SC developed a unique model of care teams composed of nurses, midwives, obstetricians, psychiatrists, diabetic educators, social workers, therapists, and lawyers. Through this intervention patients received support in accessing benefits, leaving violent relationships, lactation support services, and mental health counseling.

**Fig. 1:**
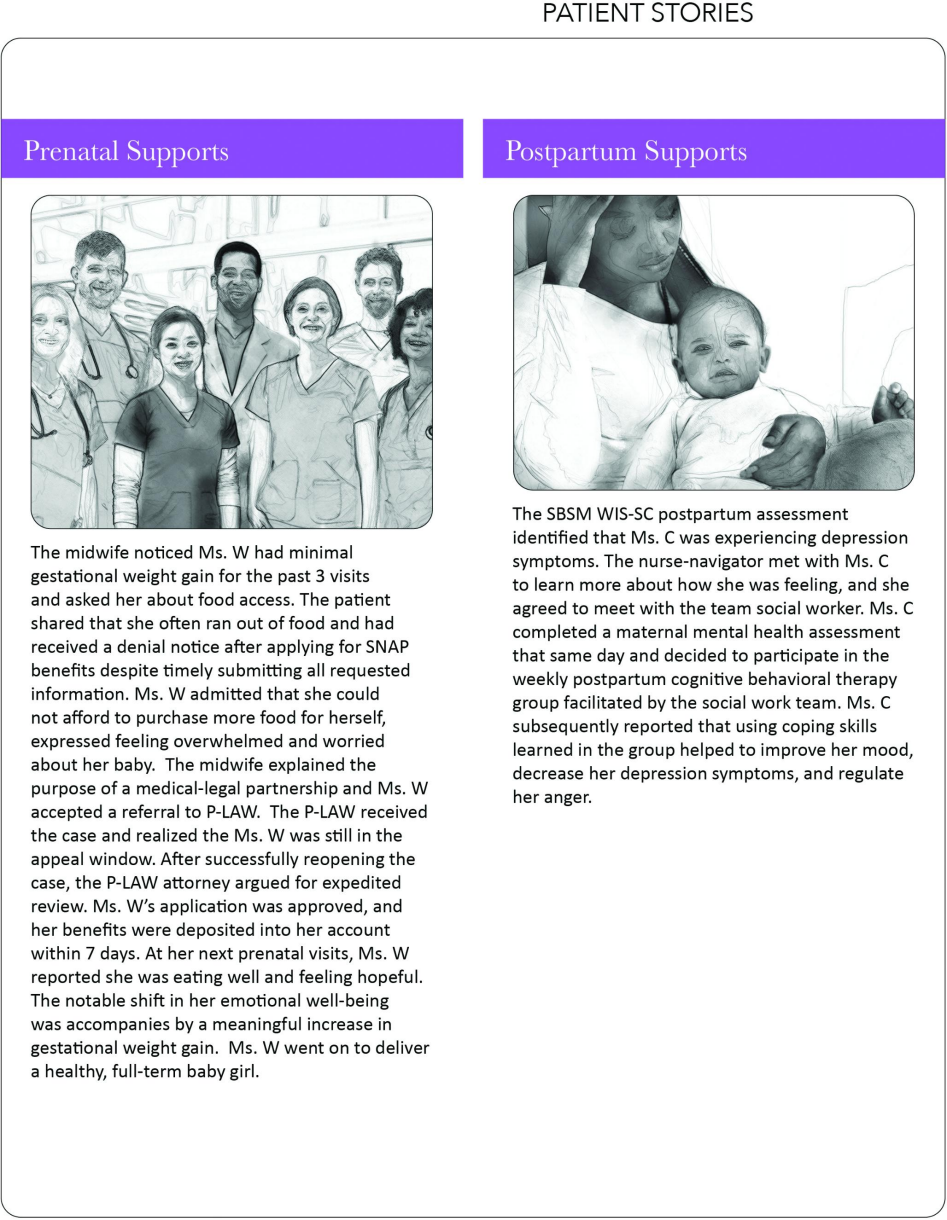
SBSM WIS-SC provides interdisciplinary, collaborative care grounded in a trauma informed approach

Outcome data on the impact of these services on maternal and child health outcomes are still under evaluation; however, patient case studies (Fig. 1) reflect positive changes at the individual level in relation to patients accessing critical services for themselves and their families. These stories reflect the complex and interrelated needs of patients from housing and food insecurity to mental health. Transformative care models, like SBSM, are urgently needed to address these multifaceted health needs and to counter the harmful effects of structural racism in health care.

## Data Availability

Data available upon reasonable request and in compliance with MedStar Health Researh Institute IRB guidelines.
